# Surveillance of foodborne parasitic diseases in Europe in a One Health approach

**DOI:** 10.1016/j.parepi.2021.e00205

**Published:** 2021-02-03

**Authors:** Joke van der Giessen, Gunita Deksne, Maria Angeles Gómez-Morales, Karin Troell, Jacinto Gomes, Smaragda Sotiraki, Miroslaw Rozycki, István Kucsera, Olgica Djurković-Djaković, Lucy J. Robertson

**Affiliations:** a National Institute for Public Health and the Environment (RIVM), Antonie van Leeuwenhoeklaan 9, 3721 MA Bilthoven, P.O. Box 1, Bilthoven 3720 BA, Netherlands; b Institute of Food Safety, Animal Health and Environment “BIOR”, Lejupes Str. 3, Riga LV-1076, Latvia; c Faculty of Biology, University of Latvia, Jelgavas Str. 1, Riga LV-1004, Latvia; d European Union Reference Laboratory for Parasites, Istituto Superiore di Sanità, Viale Regina Elena, 299, Rome 00161, Italy; e National Veterinary Institute, Ulls väg 2B, Uppsala SE-751 89, Sweden; f National Institute for Agrarian and Veterinary Research, Av. da República, Quinta do Marquês, Oeiras 2780-157, Portugal; g Veterinary Research Institute, Hellenic Agricultural Organisation-Demeter, Thermi, Thessaloniki 57001, Greece; h National Veterinary Research Institute, Aleja Partyzantów 57, Puławy 24-100, Poland; i National Public Health Center, Albert Flórián út 2-6, Budapest 1097, Hungary; j Centre of Excellence for Food- and Vector-borne Zoonoses, Institute for Medical Research, University of Belgrade, Dr. Subotića 4, Belgrade 11129, Serbia; k Department of Paraclinical Sciences, Faculty of Veterinary Medicine, Norwegian University of Life Sciences, Adamstuen Campus, Ullevålsveien 72, Oslo 0454, Norway

**Keywords:** Animals, One Health surveillance, Foodborne parasites, Underreporting

## Abstract

In 2012, WHO/FAO ranked 24 foodborne parasites (FBP) using multicriteria decision analysis (MCDA) to provide risk assessors with a basis for prioritising control of highly ranked FBP on the global level. One conclusion was that ranking may differ substantially per region. In Europe, the same methodology was used to rank FBP of relevance for Europe. Of the 24 FBP, the top-five prioritised FBP were identified for Europe as *Echinococcus multilocularis*, *Toxoplasma gondii*, *Trichinella spiralis*, *E. granulosus*, and *Cryptosporidium* spp., all of which are zoonotic. The objective of the present study was to provide an overview of surveillance and reporting systems in Europe for these top five prioritised FBP in the human and animal populations, to identify gaps, and give recommendations for improvement. Information on the surveillance systems was collected from 35 European countries and analysed according to the five different regions. For most FBP, human surveillance is passive in most countries and regions in Europe and notification differs between countries and regions. Adequate surveillance programmes for these FBP are lacking, except for *T. spiralis*, which is notifiable in 34 countries with active surveillance in susceptible animals under EU directive. Although human and animal surveillance data are available for the five prioritised FBP, we identified a lack of consistency in surveillance and reporting requirements between national experts and European bodies. Recommendations for improved surveillance systems are discussed.

## 1 Introduction

Foodborne parasites (FBP) have long been neglected, mainly due to their complex life cycles, various transmission routes, and often chronic health effects (Robertson, 2018). Using risk-ranking methods such as multicriteria decision analyses (MCDA) and disease-burden estimations (Devleesschauwer et al., 2017), FBP have been increasingly recognised as being responsible for considerable disease burdens globally (Havelaar et al., 2015). In 2012, FAO/WHO ranked 24 FBP according to MCDA, to provide risk assessors with a basis for prioritising control of highly ranked FBP (Food and Agriculture Organization of the United Nations/World Health Organization (FAO/WHO), 2014). *Taenia solium*, a cestode, ranked highest of the 24 FBP on the global level using MCDA. In addition, the WHO Foodborne Disease Burden Epidemiology Reference Group (FERG) estimated the global disease burden using the disability-adjusted life years (DALYs) metric for 31 foodborne microbiological and chemical hazards. Among FBP, *T. solium* was found to be responsible for the greatest burden of DALYs (Torgerson et al., 2015) indicating that both risk-ranking methods identified the same FBP on the global level as most important. One conclusion of the FAO/WHO global-perspective exercise was that ranking may differ substantially by region. During COST Action FA1403, A European Network for Foodborne Parasites (Euro-FBP), an approach similar to that of FAO/WHO was used to determine and rank the most important FBP in Europe (Bouwknegt et al., 2018). Based on MCDA, *Echinococcus multilocularis* was the highest-ranked FBP in Europe, followed by, in order, *Toxoplasma gondii*, *Trichinella spiralis*, *E. granulosus*, and *Cryptosporidium* spp. (Bouwknegt et al., 2018).

The cestode *E. multilocularis*, which ranked the highest in Europe, is the aetiological agent of alveolar echinococcosis (AE), a severe zoonotic disease with a substantial impact on human health, and among the most important emerging parasitic diseases in Europe (Hegglin and Deplazes, 2013; European Food Safety Agency Panel on Biological Hazards (BIOHAZ) et al., 2018). Humans become infected through ingestion of the tapeworm eggs shed by the definitive host, mainly foxes in Europe. A source-attribution systematic review and meta-analysis indicated that both drinking water and food, as well as direct contact with infected hosts, were relevant transmission vehicles (Torgerson et al., 2020). AE is commonly associated with a long incubation period that may last for more than ten years (Conraths et al., 2017). The second-highest ranked parasite in Europe, the zoonotic protozoan *T. gondii*, causes congenital and acquired toxoplasmosis. Humans acquire *T. gondii* infection through consumption of raw/undercooked meat containing viable tissue cysts, or through ingestion of oocysts in soil, water or as contaminants of fresh produce (European Food Safety Agency Panel on Biological Hazards (BIOHAZ) et al., 2018). *T. spiralis*, the third-ranked FBP, is a zoonotic nematode causing trichinellosis when raw or undercooked meat from an infected animal is consumed. Although the disease burden of trichinellosis is considered of minor importance at the global level (Havelaar et al., 2015) and ranked 7th on the global scale using MCDA, in Europe the situation is different. Almost half of all reported cases worldwide are reported in Europe (Devleesschauwer et al., 2015) where domestic pigs not raised under controlled housing conditions and wild boar are the main sources of human infections. *T. spiralis* (and other *Trichinella* species) are the only FBP of these top-five for which there is mandatory-targeted surveillance in susceptible meat-producing animals that are placed on the EU market during meat inspection in Europe (European Commission, 2015). An exemption has recently been implemented for fattening pigs raised under controlled housing conditions (European Commission, 2015). Tapeworms of the *E. granulosus* complex cause cystic echinococcosis (CE) in humans through ingestion of the eggs shed by the definitive host, mainly dogs. Since the incubation period is long and can range from five to fifteen years, risk factors are difficult to identify although contaminated raw produce or water are considered the transmission pathway (Possenti et al., 2016). This zoonotic fourth-ranked FBP can cause economic losses in animals and high morbidity in people (Torgerson et al., 2015; European Food Safety Agency Panel on Biological Hazards (BIOHAZ) et al., 2018). CE has a lower disease burden than AE due to lower mortality, but exerts a substantial burden in southern Europe (Tamarozzi et al., 2018). The fifth-ranked parasite in Europe was the protozoan *Cryptosporidium* spp. At least 30 species of *Cryptosporidium* have been described; some are host specific, whereas others can infect various hosts. *Cryptosporidium parvum* (zoonotic) and *C. hominis* are the species most commonly associated with human infection in Europe, after oral uptake of oocysts shed by infected hosts. The main risk factors for *C. hominis* are linked to contact with young children, people with diarrhoea or contamination of water by human waste or wastewater. Although *C. parvum* can also be spread between people, the main risk factors are linked to contact with farm animals, especially young stock (e.g., at petting farms), or consumption of water or food contaminated by their faeces (Cacciò and Chalmers, 2016). Cryptosporidiosis causes self-limiting diarrhoea in immunocompetent hosts (European Food Safety Agency Panel on Biological Hazards (BIOHAZ) et al., 2018; Cacciò and Chalmers, 2016).

The European One Health zoonoses report, published annually by European Food Safety Authority in Parma (EFSA) and European Center for Disases Control in Stockholm (ECDC), describes data on zoonoses, zoonotic agents, antimicrobial resistance and food-borne outbreaks (FBO) under the Zoonoses Directive 2003/99/EC (Community, 2003). Among the top-five prioritised FBP, it is mandatory to report data on trichinellosis and echinococcosis; for cryptosporidiosis and toxoplasmosis, reporting is based on national considerations (European Food Safety Authority and European Centre for Disease Prevention and Control, 2018). The accuracy, reliability, and usefulness of these data depend on systematic and uniform surveillance and reporting across Europe. However, we know that this is not always the case.

Surveillance systems may be implemented for different purposes such as early warning and detection, clinical case finding, to estimate the magnitude or occurrence of infection by prevalence or incidence, or to determine freedom of disease. In animal populations, surveillance could also serve to prevent human infections. One example is mandatory slaughterhouse surveillance for *Trichinella*, where positive animals are removed from the food chain, thereby preventing human infections. Clinical case reporting is commonly used in human surveillance and an example of passive surveillance. In active surveillance, analyses for a particular pathogen in human or animal populations are conducted following a specified sampling plan. Risk-based surveillance and surveillance systems to determine freedom of disease are increasingly being used in veterinary medicine (Stärk et al., 2016). Risk-based surveillance and control principles for FBP have recently been described by Alban et al. (2020), whereas the control of *Trichinella* spp. in Suidae in Europe, has been regulated at the EU level. The advantage of risk-based surveillance is increased cost effectiveness of the surveillance (European Commission, 2015).

The present study provides an overview of the different surveillance and reporting systems in Europe for the top-five prioritised FBP in human and animal populations, identifies gaps, and suggests recommendations for improvement.

## 2 Materials and methods

The survey was conducted in the same five European regions as reported for prioritising FBP in Europe: Northern, Western, Eastern, Southeastern, and Southwestern as previously described (Bouwknegt et al., 2018). Regional coordinators of the Euro-FBP network identified and contacted experts or agencies in each country of their region, sending them a questionnaire for completion and a glossary document with explanations. The questionnaire requested information about disease notification and surveillance systems for humans and animals during the previous five years (2014–2018), the populations (for humans) under surveillance, reporting sources, and case definitions as described in EU decision 2018/945 (Commission Implementing Decision (EU), 2018). Data of the various countries per parasite were combined in a common Excel database and afterwards analysed per region. Results from the different regions were combined per parasite and discussed in a common meeting of all regional coordinators. An additional online questionnaire was subsequently used to address gaps and inconsistencies identified at the meeting (Supplementary file). We did not wish to include project-based surveillance, as this may be non-systematic and short-term, unless conducted according to governmental requirements (e.g., *E. multilocularis* in wildlife to document freedom from infection).

The information collected used the following pre-agreed definitions and criteria:

● Notifiable: by law of that country.
● Passive surveillance: gathering data from clinical or laboratory diagnosed cases of infection.
  ● For humans, notifiable diseases are based on passive surveillance of the whole population; for non-notifiable diseases, this can be for a restricted population (e.g., pregnant women).
  ● For animals, only for infections that cause clinical symptoms, such as *E. multilocularis* infections in dogs causing alveolar echinococcosis; *T. gondii* infections in pregnant sheep, *Cryptosporidium* infection in ruminants.
● Active surveillance: analyses for a particular pathogen in human or animal populations follow a statistical or probability-based sampling plan. This is important in surveillance of diseases in which subclinical cases/carriers predominate. Active surveillance in humans may be population-based for, e.g., government records. Data from meat inspection of slaughtered animals for food production in the abattoir is defined as active surveillance in this study. Population-based surveillance can also include monitoring for justifying derogation from regulations, such as compulsory treatment of imported dogs into countries with proven absence of *E. multilocularis* in wildlife (Commission Delegated Regulation (EU), 2018; European Food Safety Authority and European Centre for Disease Prevention and Control, 2019).

The various active and passive surveillance systems in the different countries were not evaluated for their effectiveness, and no data on this aspect were requested in the questionnaire.

## 3 Results

### 3.1 General overview

In total, 35 countries including EU Member States (MS) and non-MS in five European regions were included in the study.

• In Northern (N) Europe: Denmark (DK), Finland (FI), Iceland (ICE), Norway (NO), Sweden (SWE).
• In Western (W) Europe: Austria (AU), Belgium (BE), Germany (DE), France (FR), Liechtenstein (LI), Republic of Ireland (IE), The Netherlands (NL), Switzerland (CH), United Kingdom (UK).
• In Eastern (E) Europe: Czech Republic (CZ), Estonia (EE), Hungary (HU), Latvia (LV), Poland (PL), Romania (RO), Slovakia (SK).
• In Southwestern (SW) Europe: Italy (IT), Portugal (PT), Spain (ES).
• In Southeastern (SE) Europe: Albania (AL), Bosnia and Herzegovina (BH); Bulgaria (BG); Croatia (HR), Cyprus (CY), Greece (GR), Montenegro (ME), North Macedonia (MK), Serbia (RS), Slovenia (SL), Turkey (TR).

For many countries, information was obtained from public health and animal health institutes and food-safety agencies. The different surveillance systems in humans and animals are combined by region (Table 1, Table 2, Table 3, Table 4, Table 5), and also described by parasite for all regions.

**Table 1:** Surveillance systems in humans and animals in Northern Europe. Countries included in the region – Denmark (DK), Finland (FI), Iceland (ICE), Norway (NO), Sweden (SWE).

Disease/parasitic agent	Human			Animals			
	Notifiable	Active surveillance	Passive surveillance	Notifiable	Active surveillance	Population under active surveillance	Passive surveillance
Alveolar echinococcosis/*Echinococcus multilocularis*	All except DK^a^	None	All	All	DK, FI, ICE, NO^d^	Red foxes and slaughtered animals^e^	All^g^
Toxoplasmosis/*Toxoplasma gondii*	FI, ICE^b^	ICE	DK, FI, ICE	FI^c^; ICE	None	None	FI, ICE^g^
Trichinellosis/*T. spiralis* and other *Trichinella* spp.	All except DK^a^	None	All	All	All	Slaughtered pigs, solipeds, wild boar, bears^f^	None
Cystic echinococcosis/*Echinococcus granulosus*	All except DK^a^	None	All	All	All	Slaughtered animals; red foxes and dogs^e^	All^g^
Cryptosporidiosis/*Cryptosporidium* spp.	All except DK^a^	None	All	None	DK	Cervids	None

a DK is currently developing its notification system;

b FI, ICE report only congenital toxoplasmosis;

c In FI *T. gondii* is notifiable in swine, sheep, goat, dog, cat and ferret;

d FI and mainland NO confirmed the free status for *E. multilocularis* in the context of Regulation (EU) No. 1152/2013 (EFSA, 2019); in DK active surveillance is present in cervids for *Cryptosporidium* spp.;

e *E. multilocularis* and *E. granulosus* – slaughter animals under meat inspection former EU Directive 854/2004 since 13/12/2019 replaced by EU directive 2019/627 and governmental monitoring programmes for red foxes; only in ICE dogs are also under active surveillance for *E. granulosus*;

f *Trichinella* spp. – mandatory surveillance/control of slaughtered pigs, solipeds, wild boar and susceptible animals intended for human consumption according to EU regulation 2015/1375, in SWE also in bears.

g Passive surveillance for alveolar echinococcosis (*E. multilocularis*) in dogs and cats, toxoplasmosis (*T. gondii*) in sheep; *E. granulosus* shedding in canids. Reporting uncertain.

**Table 2:** Surveillance systems in humans and animals in Western Europe. Countries included in the region – Austria (AU); Belgium (BE); France (FR); Germany (DE); Liechtenstein (LI); Republic of Ireland (IE); The Netherlands (NL); Switzerland (CH); United Kingdom (UK).

Disease/parasitic agent	Human			Animals			
	Notifiable	Active surveillance	Passive surveillance	Notifiable	Active surveillance	Population under active surveillance	Passive surveillance
Alveolar echinococcosis/*Echinococcus multilocularis*	AU, DE, FR, IE	None	All	BE, CH, DE, IE, NL, UK	AU, BE, CH, DE, IE, NL, UK^c^	Red foxes and slaughtered animals^d^	BE, CH, FR, LI, NL^f^
Toxoplasmosis/*Toxoplasma gondii*	DE; FR; IE^a^, UK	AU, BE, DE, FR, NL^b^	All, except BE, NL	BE, CH, DE, IE, LI, NL	BE, DE, FR, NL	Livestock	BE, CH, DE, IE, LI, NL, UK^f^
Trichinellosis/*T. spiralis* and other *Trichinella* spp.	All except BE, FR, UK	None	All	All	All	Slaughtered pigs, solipeds, red fox, wild boar^e^	None
Cystic echinococcosis/*Echinococcus granulosus*	AU, DE, FR, IE	None	All	All	All	Slaughtered animals^d^	CH, DE, NL^f^
Cryptosporidiosis/*Cryptosporidium* spp.	DE, IE, UK	NL^g^	All	CH	None	None	CH, IE, NL, UK^f^

a Congenital toxoplasmosis;

b For toxoplasmosis in AU, BE, DE, FR – screening in pregnant women; NL and DE – seroprevalence.

c IE and UK confirm the free status for *E. multilocularis* in the context of Regulation (EU) No. 1152/2011 (EFSA 2019), seroprevalence studies in BE, DE, FR and NL reported;

d *E. multilocularis* and *E. granulosus* – slaughter animals under meat inspection former EU Directive 854/2004 since 13/12/2019 replaced by EU directive 2017/627 for cysticercosis; and governmental monitoring programmes for red foxes.

e *Trichinella* spp. – mandatory surveillance/control of slaughtered pigs, solipeds, wild boar and susceptible animals intended for human consumption according to EU regulation 2015/1375; In IE risk based active surveillance in red foxes.

f Passive surveillance for alveolar echinococcosis (*E. multilocularis*) in dogs and cats, toxoplasmosis (*T. gondii*) in sheep*; E. granulosus* shedding in canids, Cryptosporidiosis (*Cryptosporidium* spp) in ruminants. Reporting uncertain.

g Laboratory surveillance.

**Table 3:** Surveillance systems in humans and animals in Eastern Europe. Countries included in the region – Czech Republic (CZ); Estonia (EE), Hungary (HU); Latvia (LV); Poland (PL); Romania (RO), Slovakia (SK).

Disease/parasitic agent	Human			Animals			
	Notifiable	Active surveillance	Passive surveillance	Notifiable	Active surveillance	Population under active surveillance	Passive surveillance
Alveolar echinococcosis/*Echinococcus multilocularis*	All	PL	All	All	CZ; PL	Slaughtered animals; red foxes^c^	LV; PL; RO; SK^f^
Toxoplasmosis/*Toxoplasma gondii*	All^a^	CZ, PL, HU, SK	All	PL, LV	PL^b^	Cattle, pigs^b^	LV; PL; RO; SK^f^
Trichinellosis/*T. spiralis* and other *Trichinella* spp.	All	None	All	All	All	Slaughtered pigs, solipeds and wild boar; red foxes^d^	None
Cystic echinococcosis/*Echinococcus granulosus*	All	PL	All	All	CZ; HU; LV; PL; SK	Slaughtered animals; dogs, red foxes^c^	LV; PL; RO; SK^f^
Cryptosporidiosis/*Cryptosporidium* spp.	All except PL, RO, SK	None	All	None	PL^e^	Cattle^e^	CZ; LV; PL; RO; SK^f^

a Mainly congenital toxoplasmosis is notifiable, in CZ only acquired toxoplasmosis is notifiable, in HU, EE, SK – both congenital and acquired toxoplasmosis is notifiable;

b In PL seroprevalence studies in cattle and pigs;

c *E. multilocularis* and *E. granulosus* – slaughter animals under meat inspection former EU Directive 854/2004 since 13/12/2019 replaced by EU directive 2019/627 in CZ red foxes intended for rabies examination across whole country yearly, in HU 1% of red fox population every fourth year, in PL red foxes and dogs within governmental monitoring program;

d *Trichinella* spp. – mandatory surveillance/control of slaughtered pigs, solipeds, wild boar and susceptible animals intended for human consumption according to EU regulation 2015/1375, in CZ red foxes intended for rabies examination across whole country yearly, in HU 1% of red fox population every fourth year;

e *Cryptosporidium* spp. – in PL slaughtered animals under governmental monitoring program.

f Passive surveillance for alveolar echinococcosis (*E. multilocularis*) in dogs and cats; toxoplasmosis (*T. gondii*) in sheep; *E. granulosus* shedding in canids, cryptosporidiosis(*Cryptosporidium spp*.) in ruminants. Reporting uncertain.

**Table 4:** Surveillance systems in humans and animals in Southwestern Europe. Countries included in the region – Italy (IT); Portugal (PT); Spain (ES).

Disease/parasitic agent	Human			Animals			
	Notifiable	Active surveillance	Passive surveillance	Notifiable	Active surveillance	Population under active surveillance	Passive surveillance
Alveolar echinococcosis/*Echinococcus multilocularis*	All	None	All	All	All	Slaughtered animals^c^	None
Toxoplasmosis/*Toxoplasma gondii*	All^a^	All^b^	All	None	None	None	ES^e^
Trichinellosis/*T. spiralis* and other *Trichinella* spp.	All	None	All	All	All	Slaughtered pigs, solipeds, wild boar^d^	None
Cystic echinococcosis/*Echinococcus granulosus*	All	None	All	PT	All	Slaughtered animals^c^	ES, IT^e^
Cryptosporidiosis/*Cryptosporidium* spp.	ES; PT	None	All	None	None	None	None

a Congenital toxoplasmosis; in IT, toxoplasmosis is notifiable without specifying the form of infection (congenital or acquired);

b For congenital toxoplasmosis in IT, PT – screening in pregnant women; ES – only in some regions;

c *E. multilocularis* and *E. granulosus* – slaughtered animals under meat inspection former EU Directive 854/2004 since 13/12/2019 replaced by EU directive 2019/627 for cysticercosis.

d *Trichinella* spp. – mandatory surveillance/control of slaughtered pigs, solipeds, wild boar and susceptible animals intended for human consumption according to EU regulation 2015/1375.

e Passive surveillance for alveolar echinococcosis (*E. multilocularis*) in dogs and cats; toxoplasmosis (*T. gondii*) in sheep; *E. granulosus* shedding in canids, Cryptosporidiosis (*Cryptosporidium* spp) in ruminants. Reporting uncertain.

**Table 5:** Surveillance systems in humans and animals in Southeastern Europe. Countries included in the region – Albania (AL); Bosnia and Herzegovina (BH); Bulgaria (BG); Croatia (HR), Cyprus (CY), Greece (GR), North Macedonia (MK), Montenegro (ME), Serbia (RS), Slovenia (SL), Turkey (TR).

Disease/parasitic agent	Human			Animals			
	Notifiable	Active surveillance	Passive surveillance	Notifiable	Active surveillance	Population under active surveillance	Passive surveillance
Alveolar echinococcosis/*Echinococcus multilocularis*	All except BH, BG, ME	None	All	AL, CY, GR, MK, RS, SL	RS, SL	Slaughtered animals red foxes^d^	AL^f^
Toxoplasmosis/*Toxoplasma gondii*	All except TR^a^	BG, HR, SL, RS^b^	All^c^	MK, RS, SL	None	None	MK, RS, SL^f^
Trichinellosis/*T. spiralis* and other *Trichinella* spp.	All except ME, TR	BG	All except ME, TR	All except TR	All	Slaughtered pigs, solipeds, wild boar^e^	None
Cystic echinococcosis/*Echinococcus granulosus*	All	None	All	All	All except AL, BG, MK	Slaughtered animals^d^	BG, MK, SL^f^
Cryptosporidiosis/*Cryptosporidium* spp.	BG, HR, MK, ME, SL	None	All	None	None	None	MK^f^

a Congenital toxoplasmosis and for TR toxoplasmosis cases are registered but not compulsory to declare;

b For toxoplasmosis screening in pregnant women;

c In BG, CY, GR, TR passive surveillance only for congenital toxoplasmosis;

d *E. multilocularis* and *E. granulosus* – slaughter animals under meat inspection former EU Directive 854/2004 since 13/12/2019 replaced by EU directive 2017/627 for cysticercosis, red foxes by governmental monitoring programmes;

e *Trichinella* spp. – mandatory surveillance/control of slaughtered pigs, solipeds, wild boar and susceptible animals intended for human consumption according to EU regulation 2015/1375;

f Passive surveillance for alveolar echinococcosis (*E. multilocularis*) in dogs and cats; toxoplasmosis (*T. gondii*) in sheep; *E. granulosus* shedding in canids, Cryptosporidiosis (*Cryptosporidium* spp) in ruminants. Reporting uncertain.

Active surveillance during meat inspection was described in EU Regulation 854/2004 and, since 2019, replaced by EU Regulation 627/2019 (European Regulation (EC), 2004; Commission Regulation (EU), 2019) for the MS. In the EU Regulation, practical arrangements for official control for cysticercosis during post-mortem inspection caused by *Taenia saginata* in domestic bovine animals and by *T. solium* for Suidae and for *Trichinella* spp. in susceptible slaughter animals are included. Risk-based surveillance has been in place for *T. saginata* in bovine animals since 2019, and for *Trichinella* in slaughter pigs since 2015 (European Commission, 2015). For *Echinococcus* spp. no additional arrangements are included. As *Echinococcus* spp. cysts are identifiable during meat inspection, both *E. granulosus* and *E. multilocularis* lesions are included as active surveillance in Table 1, Table 2, Table 3, Table 4, Table 5 for slaughter animals. However, *E. multilocularis* cysts are seldom present in slaughter animals.

All countries reported that they follow case definitions of the parasitic diseases under survey, according to EU decision 945/2018 in all regions (Commission Implementing Decision (EU), 2018).

### 3.2 Surveillance systems per FBP in the different regions

#### 3.2.1 *Echinococcus multilocularis* and alveolar echinococcosis

Human cases of both alveolar echinococcosis (AE) caused by *E. multilocularis* and cystic echinococcosis (CE) caused by *E. granulosus* sensu lato (s.l.) are reported as echinococcosis, as the EU case definition EU Decision 2018/945 (Commission Implementing Decision (EU), 2018) does not distinguish between these diseases. Countries can, however, report cases into the European Surveillance System database by species and, since 2019 (2018 data), by clinical presentation of the disease. ECDC can use that data to differentiate between the two diseases. AU, CZ, DE, DK, FI, FR, HU, ICE, IE, NL, NO, SK and SWE declared reporting AE separately from CE. Reporting of echinococcosis differs regionally, being notifiable in most countries, but not in BE, CH, LI, NL and UK, where reporting is based on voluntary passive surveillance (European Food Safety Authority and European Centre for Disease Prevention and Control, 2019). DK is developing its notification system. The disease is also not notifiable in BH, BG and ME.

In animals, *E. multilocularis* is notifiable in six countries in W Europe (BE, CH, DE, IE, NL and UK) and all countries in N Europe and SW Europe, and six countries in SE Europe (AL, CY, GR, MK, RS, SL). In addition, *E. multilocularis* is notifiable in all countries in E Europe; in HU (where all susceptible animals are examined for *Echinococcus* and *Trichinella* at slaughterhouses) parasite isolates are sent with background data to the National Reference Laboratory (NRL) for Parasites for verification and species (genotype) identification according to the Ministry of Agriculture. In several countries (AU, BE, CH, CZ, DE, IE, NL, PL, UK and all countries in N Europe, except SWE), there is active surveillance of wildlife for *E. multilocularis* (mainly red foxes). Four countries (FI, IE, UK, and mainland NO) have demonstrated the absence of *E. multilocularis* through implementation of an annual surveillance programme required by the EU in accordance with Regulation (EU) No. 2018/772 (Commission Delegated Regulation (EU), 2018; European Food Safety Authority and European Centre for Disease Prevention and Control, 2019).

Alveolar echinococcosis in dogs and cats will be reported during passive surveillance in BE, CH, FR, LI and NL but is very rare in N and S Europe and therefore unclear if it will be reported in many regions.

Speciation of *Echinococcus* in animals is reported from CZ, DK, EE, FI, FR, HU, ICE, NL, NO, SK, and SWE.

#### 3.2.2 *Toxoplasma gondii* and toxoplasmosis

In humans, the case definition is that of the EU (Commission Implementing Decision (EU), 2018) and sometimes regulated by national legislation for acquired toxoplasmosis, as, for example, in HU and PT.

Congenital toxoplasmosis is notifiable in most European countries, except for AU, BE, CH, DK, NO, NL, and SWE, while both acquired and congenital toxoplasmosis are notifiable in AL, BH, BG, CY, EE, ES, FI, GR, HR, HU, ICE, IE, ME, MK, LV, PL, PT, RO, RS, SL and SK. In IT, toxoplasmosis is notifiable without specifying the form of infection (congenital or acquired), whereas acquired toxoplasmosis is notifiable in CZ. Screening pregnant women is mandatory in AU, BE, FR, SK, HR, IT, PL, and SL, and, since 2017, in RS, but is voluntary in BG, HU, and also in CZ and DE where screening is not covered by statutory health insurance (European Centre for Disease Prevention and Control, 2019). In ES, screening is managed at the Autonomous Communities level. In PT, screening it is not mandatory but highly recommended.

Passive surveillance of human clinical cases occurs in most countries, but whether these cases are reported is unclear. Clinical cases are not reported in BE and NL. In some countries with passive surveillance (e.g., CZ, EE, HU, SK, SR) both hospitalised and other patients with clinical signs are reported. In E Europe, active surveillance is carried out in PL in pregnant women, HIV-positive patients, and organ donors/transplant recipients. Other countries in this region report passive surveillance, but in different target groups, with mainly pregnant women and HIV-positive patients in CZ, EE, HU, LV, SK; preterm neonates and infants in HU and SK; organ donors and transplant recipients in SK; and patients with clinical signs and hospitalised patients in PL, RO, SK.

Population-based serosurveillance studies have been reported from AU, BE, DE, ES, FR, ICE, NL, NO, PT and SWE (Hofhuis et al., 2011; Findal et al., 2015; Evengård et al., 2001; Birgisdóttir et al., 2006). Underreporting in most countries seems likely due to the lack of clear rules for reporting.

In animals, toxoplasmosis is notifiable in BE, CH, DE, FI, FR, ICE, IE, LI, LV, NL, MK, PL, RS and SL. Passive surveillance based on clinical cases in N Europe occurs only in ICE and FI. In W Europe, infections (abortions in small ruminants) are notifiable in BE, IE, and NL. Toxoplasmosis is reportable in livestock, companion animals, and zoo animals in CH, DE, and LI, but whether these cases are reported is unclear. Passive surveillance is carried out in LV, PL, RO, and SK in all animal species with clinical signs and/or to determine cause of death. In several countries in SE Europe, clinical cases (abortions) in sheep and goats are notifiable, and passive surveillance based on differential diagnosis in aborted small ruminants occurs in some Autonomous Communities in ES.

In some countries, active surveillance is carried out (serology and molecular methods) in slaughtered pigs, cattle, and sheep. However, these programmes are not intended as control measures (European Food Safety Agency Panel on Biological Hazards (BIOHAZ) et al., 2018).

#### 3.2.3 *Trichinella spiralis* and trichinellosis

Notification of trichinellosis in humans is mandatory in all countries, except BE, FR, ME, TR and UK that have voluntary surveillance. DK also does not report surveillance.

In all regions and almost all countries, *Trichinella* infection in animals is notifiable. Active surveillance is carried out in all countries during meat inspection according to EC regulation 1375/2015 (European Commission, 2015; Commission Regulation (EU), 2019). In IE, there is (active) risk-based surveillance in wildlife (red foxes); in PT there is an official surveillance plan for hunted wild boar, and in ES and IT there is serological follow up in positive pig farms. In CZ, four red foxes are examined per 100 km^2^ annually, and active surveillance of wild boars and other wild animals (e.g. beavers, lynx, bear) used for human consumption is reported.

#### 3.2.4 *Echinococcus granulosus* and cystic echinococcosis

CE is notifiable in most countries in Europe, except BE, CH, DK, LI, NL, and UK, but there is no active surveillance. The case definition of EU decision 2018/945 is used by those countries where CE is notifiable, but with no separate case definitions for AE and CE and speciation of human cases as previously described.

In most countries, passive surveillance is based on reporting clinical cases. In ES, passive surveillance is conducted through the “Red Nacional de Vigilancia Epidemiológica”. In IT, a register of CE that contains information from clinical cases has been extended to the European Register of Cystic Echinococcosis (Rossi et al., 2016), to collect harmonised clinical data in the EU on a voluntary basis.

In animals, *E. granulosus* is notifiable in most countries except IT and ES. Active surveillance is mandatory in all countries by visual inspection of cysts in liver and lungs during meat inspection of ruminants, horses, and other susceptible animals, according to European Regulation 2019/627 (Commission Regulation (EU), 2019). PCR confirmation of suspected cysts is reported by FR and NL.

#### 3.2.5 *Cryptosporidium* spp. and cryptosporidiosis

Human surveillance is passive in almost all countries and regions of Europe. There is wide variation both within and among regions regarding notification of cases, with cryptosporidiosis being notifiable in most countries in N Europe except DK, three countries in W Europe (DE, IE, UK), all countries in E Europe except PL, RO, and SK, all countries in SW Europe except IT, and five countries (BG, HR, MK, ME, SL) in SE Europe. Most countries report all diagnosed patients with clinical signs, but SK reports only hospitalised cases. Some countries (CZ, DK, FR, NL, NO, SWE) report that they identify and report specific *Cryptosporidium* species. However, there are differences regarding *Cryptosporidium* speciation: in SWE cases are speciated at the public health agency in the summer period and during outbreaks. In NO speciation is sometimes conducted, particularly during outbreak investigations.

Animal surveillance for *Cryptosporidium* infection is passive (animals presenting with clinical signs) in all countries, apart from active surveillance of cervids in DK and cattle in PL. *Cryptosporidium* infection in animals is not notifiable in any country except in CH. Some countries (CZ, PL, FR, DK, NL) report specific *Cryptosporidium* species to EFSA.

## 4 Discussion

Here we provide an overview of different surveillance systems in Europe for the top-five prioritised FBP in human and animal populations. These FBP, all of which are zoonotic, were prioritised using a similar approach as that used by WHO/FAO on the global scale, and based on different, mostly public health-based, criteria (Bouwknegt et al., 2018). Among these five FBP, infections caused by *E. multilocularis*, *E. granulosus*, and *T. gondii* tend not to cause acute clinical disease. For instance, most *T. gondii* infections are asymptomatic or only cause mild or unspecific symptoms; long-term sequelae, such as ocular toxoplasmosis, can occur years later. CE and AE have long incubation periods, ranging from five to fifteen years, making it extremely difficult to study outbreak situations or determine source attribution for sporadic cases. For cryptosporidiosis, with a relatively short incubation period (5–7 days), outbreaks can be more readily noticed and reported (European Food Safety Agency Panel on Biological Hazards (BIOHAZ) et al., 2018). However, even for *Cryptosporidium*, reporting is a problem in many countries due to lack of routine diagnostics and speciation of human and animal cases.

In general, although human and animal surveillance data are available for all five FBP, the surveillance and reporting requirements vary among and within regions and countries, and among national experts and European bodies.

For instance, AE in humans, which ranked as the most important FBP (Bouwknegt et al., 2018), is notifiable in N, E, and SE Europe, but in only four countries in W Europe. Moreover, echinococcosis speciation is not routinely conducted and, therefore, when human cases are diagnosed, many countries report only “echinococcosis”, despite major differences in pathology, epidemiology, and disease progress between CE and AE. Underreporting seems to be a major issue for CE in SE Europe, according to a cross-sectional study (Tamarozzi et al., 2018), ultrasound screening of people in rural areas in Bulgaria, Romania and Turkey showed a prevalence of abdominal CE of 0.41% in Bulgaria and Romania and 0.59% in Turkey. Bulgaria accounted for 26% of all confirmed echinococcosis cases reported to ECDC in 2017 and for 53% of the *E. granulosus* cases, Romania only for 2%. The incidence data reported to ECDC differed from these values by a factor of 10 for Bulgaria and a factor 700 for Romania (Tamarozzi et al., 2018). Similar results have been shown in Italy and Greece comparing research data and ECDC reports (European Food Safety Authority and European Centre for Disease Prevention and Control, 2013; Brundu et al., 2014). In animals, *Echinococcus* cysts (mainly *E. granulosus*) are not mentioned in EU regulation 2019/627 (Commission Regulation (EU), 2019) for the control of animal products intended for human consumption, although the public health impact is huge in some regions in Europe. Consequently, *E. granulosus* in slaughter animals is underreported in many countries due to the low sensitivity of meat inspection, the lack of confirmation and PCR-based speciation of suspected lesions, and an absence of data registration systems. Due to the many neglected CE cases in SW and SE Europe (Tamarozzi et al., 2018; Brundu et al., 2014), improved veterinary public health control measures are particularly needed in these regions to prevent human exposure. Effectiveness of the improved control measures can be monitored by active surveillance of the human population.

Active surveillance for *E. multilocularis* in wildlife (red foxes) occurs in many countries, but often only on voluntary basis, despite EFSA recommending harmonised surveillance in wildlife (Boué et al., 2010). Thus, understanding the prevalence and spread of this FBP is difficult. Furthermore, clinical cases in humans and infections in animals are not notifiable in many countries, and, consequently, there is substantial underreporting of this emerging parasite in Europe (Conraths et al., 2017). In conclusion, surveillance of both AE and CE in humans and animals is variable and fragmentary, with potential underreporting. In addition, suspected human cases are not confirmed by PCR in many countries, and potential *E. granulosus* findings during meat inspection are often not confirmed by PCR nor reported. Whereas *E. multilocularis* is a concern in N Europe according to the ranking as reported by Bouwknegt et al. (2018), CE is considered to be a major neglected zoonosis in S Europe with significant economic losses in the public health sector (Piseddu et al., 2017). Here the life cycle of *E. granulosus* s.s. continues, the disease burden is substantial, and improved veterinary control is needed.

Mandatory and separate notification of *E. granulosus* and *E. multilocularis* infections in both humans and relevant animals is strongly recommended in all countries, such that clearer insights into the extent of the problem are obtained and trends can be analysed.

Congenital toxoplasmosis is notifiable in 29 of the 35 countries and pregnant women are screened in some countries; nevertheless, underreporting is still a major problem in many countries. Sero-surveillance studies have been conducted in DE, ICE, ES, NL, IT, NO, PT, and SW. Data from these studies in NL has shown that toxoplasmosis has one of the highest disease burdens among foodborne diseases (Havelaar et al., 2015). Although (sero)positivity is reported in slaughtered animals in most countries, there is no mandatory control in Europe to prevent human infections via consumption of meat from infected livestock. This reinforces the need for risk-based surveillance systems in livestock. A social cost-benefit analysis of two interventions in NL showed that freezing raw meat products would be beneficial (Suijkerbuijk et al., 2019).

As trichinellosis is notifiable in humans in most countries and there is mandatory control in animals, surveillance and reporting of *T. spiralis* infections in humans and animals are the best of all five prioritised FBP. However, although serious clinical cases are probably diagnosed, mild cases may be missed because active surveillance is lacking and outbreaks continue to occur, mainly associated with meat products from pigs reared under non-controlled housing conditions and hunted wild boar. It has recently been shown that pigs from controlled housing systems in Europe pose a negligible risk, and control should be focussed on pigs reared outdoors and on wildlife intended for human consumption (Franssen et al., 2018). The EU directive 2015/1375 includes the possibility of this risk-based surveillance (European Commission, 2015). Such risk-based surveillance will not focus on pigs in controlled housing with negligible risk, but on animal populations with a high risk for *Trichinella*, such as pigs reared outdoors and wildlife intended for human consumption, such as wild boar.

Although there is general concordance across Europe regarding passive surveillance of both humans and animals for *Cryptosporidium*, there are considerable differences in reporting, resulting in a skewed impression of the distribution of this parasite. Several countries claimed to have voluntary notification, but it is unclear what such notification entails, and how the data are recorded or to whom they are accessible. Information regarding speciation of *Cryptosporidium* was also variable among and within regions; in some cases, it appeared that responsible agencies did not have a clear overview of whether speciation was conducted when cases were diagnosed. This obviously affects the data quality. Although outbreaks of cryptosporidiosis are likely to be identified due to the acute disease onset, underreporting of cases during outbreaks occurs, while many sporadic cases are probably not diagnosed.

Based on this data gathering, recommendations regarding surveillance and reporting of the top-five FBP in Europe may be defined. Improved diagnosis and reporting of human CE and AE cases in Europe is needed with emphasis on the highly endemic countries in southern Europe. Special attention should be given to species identification of the different echinococcus species by molecular methods. In addition to human CE, also the reporting and the speciation of *E. granulosus* cysts mandatory during animal slaughterhouse surveillance should be improved, and early warning and surveillance systems to determine the prevalence need to be harmonised in wildlife for *E. multilocularis* throughout Europe. EFSA (2007) recommended a harmonised surveillance scheme in wildlife with estimated prevalence sizes of targeted wildlife species, harmonised detection methods and targeted regions for surveillance throughout Europe (Boué et al., 2010), but so far surveillance has often been project based and fragmented in the different countries, making comparisons among countries extremely difficult. *T. gondii* causes is an important foodborne infection that ranked high in Europe according to the MCDA methods (Bouwknegt et al., 2018) and DALY estimates for toxoplasmosis (Havelaar et al., 2015). However, reporting of congenital toxoplasmosis is absent in many countries in Europe. More efforts are needed to obtain better insight into congenital toxoplasmosis cases and the seroprevalence in Europe to improve estimation of the disease burden and thus adequately prioritize the control measures to be taken. In animals, surveillance is underdeveloped in livestock species and risk-based surveillance of livestock needs to be improved to reduce human meat-borne infections. Risk-based surveillance in meat producing animals can be based on risk assessment studies that are available for Italy and the Netherlands (European Food Safety Agency Panel on Biological Hazards (BIOHAZ) et al., 2018) and could be used to prevent human infections and thus reduce the disease burden in humans. Trichinellosis is the only FBP where reporting is mandatory in most countries in humans, despite a low global disease burden (Torgerson et al., 2015). Therefore, how much effort is needed to control this FBP in animal populations is debatable. However, half of the reported global human cases occur in Europe(Devleesschauwer et al., 2015). Nowadays, a risk-based surveillance system can be implemented under EU reg. 2015/1375 (European Commission, 2015), which is also harmonised with guidelines for the Codex Alimentarius and the OIE (Alimentarius, 2015). Despite this, in almost all countries, all slaughtered pigs originating from controlled housing are tested. Therefore, risk-based surveillance in animals should be given more priority to be implemented in Europe to improve cost effectiveness. Improvement of human cryptosporidiosis reporting is needed, because only limited data are available about the presence and prevalence of cryptosporidiosis in human populations. Moreover, harmonised detection methods will improve comparison of reporting between countries. Determination of whether human infections are *C. parvum, C. hominis*, or another species is recommended.

## Declaration of Competing Interest

Authors declare that they have no conflict of interest.

## Appendix A Supplementary data

Supplementary material Image 1
