# Supplementary material for: Surveillance of foodborne parasitic diseases in Europe in a One Health approach
Source: Parasite Epidemiol Control. 2021 Feb 3;13:e00205. doi: 10.1016/j.parepi.2021.e00205 (PMC7900597; doi:10.1016/j.parepi.2021.e00205)
Supplement: Supplementary file 1 — Supplementary material [file mmc1.docx]

**Supplementary file 1**

**Questionnaire 1.** Description of the surveillance systems in humans and animals for the top five prioritised foodborne parasites per country in Europe.

1. Specify which of the following activities has been undertaken in your country:

| **Pathogen** | **Human health** | | | **Animal health** | | |
| --- | --- | --- | --- | --- | --- | --- |
|  | Is the pathogen NOTIFIABLE in your country? | Is there an ongoing PASSIVE surveillance? | Is there an ongoing ACTIVE surveillance? | Is this pathogen NOTIFIEABLE in your country? | Is there an ongoing PASSIVE surveillance? | Is there an ongoing ACTIVE surveillance? |
| *Toxoplasma gondii* | YES/NO | YES/NO | YES/NO | YES/NO | YES/NO | YES/NO |
| *Echinococcus granulosus* | YES/NO | YES/NO | YES/NO | YES/NO | YES/NO | YES/NO |
| *Echinococcus multilocularis* | YES/NO | YES/NO | YES/NO | YES/NO | YES/NO | YES/NO |
| *Trichinella spiralis* | YES/NO | YES/NO | YES/NO | YES/NO | YES/NO | YES/NO |
| *Cryptosporidium spp.* | YES/NO | YES/NO | YES/NO | YES/NO | YES/NO | YES/NO |
| *Other Trichinella species* | YES/NO | YES/NO | YES/NO | YES/NO | YES/NO | YES/NO |

1. Specify the population (for humans) under surveillance:

| **Pathogen** | **PASSIVE surveillance** | **ACTIVE surveillance** |
| --- | --- | --- |
| *Toxoplasma gondii* |  |  |
| *Echinococcus granulosus* |  |  |
| *Echinococcus multilocularis* |  |  |
| *Trichinella spiralis* |  |  |
| *Cryptosporidium spp.* |  |  |
| *Other Trichinella species* |  |  |

1. Specify the reporting sources for human diagnosed cases:

| **Pathogen** | **PASSIVE surveillance** | **ACTIVE surveillance** |
| --- | --- | --- |
| *Toxoplasma gondii* |  |  |
| *Echinococcus granulosus* |  |  |
| *Echinococcus multilocularis* |  |  |
| *Trichinella spiralis* |  |  |
| *Cryptosporidium spp.* |  |  |
| *Other Trichinella species* |  |  |

1. Specify the population (for animals) under surveillance:

| **Pathogen** | **PASSIVE surveillance** | **ACTIVE surveillance** |
| --- | --- | --- |
| *Toxoplasma gondii* |  |  |
| *Echinococcus granulosus* |  |  |
| *Echinococcus multilocularis* |  |  |
| *Trichinella spiralis* |  |  |
| *Cryptosporidium spp.* |  |  |
| *Other Trichinella species* |  |  |

1. Specify the reporting sources for animal infection cases:

| **Pathogen** | **PASSIVE surveillance** | **ACTIVE surveillance** |
| --- | --- | --- |
| *Toxoplasma gondii* |  |  |
| *Echinococcus granulosus* |  |  |
| *Echinococcus multilocularis* |  |  |
| *Trichinella spiralis* |  |  |
| *Cryptosporidium spp.* |  |  |
| *Other Trichinella species* |  |  |

**Questionnaire 2.** Additional questionnaire for top five ranked foodborne parasite surveillance systems to specify the used case definitions, origin of officially reported human cases under passive surveillance, and to specify if the country distinguish the species when specific pathogens are reported in humans and animal.

1. Please, specify the field you represent (select one response):
   1. Public health (continue with next questions, except 4 and 6)
   2. Animal health (continue with question 4 and 6 only)
   3. Both (continue with all questions)
   4. Other. Please, specify_______________________________
2. Within previous questionnaire, it has been noted that Toxoplasmosis in humans is notifiable disease in your country. Could you please specify which of the further mentioned diseases does your country report (select one response)?
   1. Acquired toxoplasmosis
   2. Congenital toxoplasmosis
   3. Both
   4. Toxoplasmosis is not notifiable in my country
3. Within previous questionnaire, it has been noted that there is passive surveillance of patients with clinical signs and/or hospitalized patients for toxoplasmosis, echinococcosis, cryptosporidiosis, trichinellosis. Do all those cases are also officially reported (select multiple response)?
   1. Only cases when patients diagnosed toxoplasmosis with clinical signs but not hospitalized are reported
   2. Only hospitalized toxoplasmosis cases are reported
   3. Both (hospitalized and patients with clinical signs) toxoplasmosis cases are reported
   4. Only cases when patients diagnosed echinococcosis with clinical signs but not hospitalized are reported
   5. Only hospitalized echinococcosis cases are reported
   6. Both (hospitalized and patients with clinical signs) echinococcosis cases are reported
   7. Only cases when patients diagnosed cryptosporidiosis with clinical signs but not hospitalized are reported
   8. Only hospitalized cryptosporidiosis cases are reported
   9. Both (hospitalized and patients with clinical signs) cryptosporidiosis cases are reported
   10. Only cases when patients diagnosed trichinellosis with clinical signs but not hospitalized are reported
   11. Only hospitalized trichinellosis cases are reported
   12. Both (hospitalized and patients with clinical signs) trichinellosis cases are reported
   13. Other. please, specify _____________________________________
4. Does your country distinguish the species when echinococcosis in animals is reported (select one response)?
   1. Yes
   2. No
5. Does your country distinguish the species when alveolar and/or cystic echinococcosis in humans is reported (select one response)?
   1. Yes
   2. No
6. Does your country distinguish the species when cryptosporidiosis in humans is reported (select one response)?
   1. Yes
   2. No
7. Does your country distinguish the species when cryptosporidiosis in animals is reported (select one response)?
   1. Yes
   2. No
8. What kind of case definition you use for toxoplasmosis in humans (select one response)?
   1. Following the EU Decision 2012/506
   2. Other. please, specify_______________________________________
9. What kind of case definition you use for echinococcosis in humans (select one response)?
   1. Following the EU Decision 2012/506
   2. Other. please, specify_______________________________________
10. What kind of case definition you use for cryptosporidiosis in humans (select one response)?
    1. Following the EU Decision 2012/506
    2. Other. please, specify_______________________________________
11. What kind of case definition you use for trichinellosis in humans (select one response)?
    1. Following the EU Decision 2012/506
    2. Other. please, specify_______________________________________
